# Use of a Silicone Mask Improves the Reproducibility of Tooth Color Assessment for Whitening Clinical Trials

**DOI:** 10.1155/ijod/6221075

**Published:** 2025-04-09

**Authors:** Pier Francesco Porciani, Simone Grandini, Caterina Perra, Luca Porcu, Valter Torri, Luisa Diomede, Andrea Sarrica

**Affiliations:** ^1^Department of Medical Biotechnologies, Unit of Endodontics and Restorative Dentistry, University of Siena, Viale Bracci 16, Siena, Italy; ^2^Department of Clinical Oncology, Istituto di Ricerche Farmacologiche Mario Negri IRCCS, Via Mario Negri 2 20156, Milan, Italy; ^3^Department of Molecular Biochemistry and Pharmacology, Istituto di Ricerche Farmacologiche Mario Negri IRCCS, Via Mario Negri 2 20156, Milan, Italy; ^4^R & D Department, Perfetti Van Melle SpA, XXV Aprile 7 20045, Lainate, Milan, Italy

**Keywords:** color, mask, spectrophotometer, tooth, whitening, WIO

## Abstract

**Objective:** This study aimed to investigate the reproducibility of tooth color assessment by dentists using two different spectrophotometers, Rayplicker and Vita Easyshade Advance 4.0, with or without a silicone mask developed to place its tip in the same position.

**Methods:** Twenty subjects participated in this cross-over study. Multiple color coordinates and correlated whitening index measurements were performed to compare the variability with Vita Easyshade, with or without the mask, or the Rayplicker. The intrasubject variability was evaluated in consecutive measures and over time.

**Results:** In consecutive measures, each subject's variance and the percentage of the coefficient of variation (CV) were minimal for each score. Bartlett's test resulted in a more significant variance for Vita Easyshade with a mask compared with either Vita Easyshade without a mask or Rayplicker. The comparison of the values obtained before and after 2'30” indicated complete reproducibility in variance and %CV data. *p* Values associated with the Bartlett test resulted in >0.90 for all the pre–post score comparisons, and no difference in pre–post %CV values was detected. Bartlett's test indicated that the measures with Vita Easyshade without the mask had significantly greater CV for *L* and *b* scores than those obtained with the mask or Rayplicker, which were similar among them.

**Conclusion:** This study indicates that the silicone mask improves the precision of Vita Easyshade, and Rayplicker can be a valid alternative for studies measuring the changes in tooth color after whitening treatments.

## 1. Introduction

The increasing demand for solutions to improve the appearance of white teeth has led to a greater emphasis on methods for objectively measuring tooth whiteness. Commercially available visual guides that dental practitioners use to select the porcelain prosthetic material to match the natural color of the teeth are also employed by dental practitioners and hygienists to assess whether cosmetic whitening products have a noticeable whitening effect [1, 2]. The Vita shade guide is the first and is still the most widely used tool. It was initially designed in the 1920s to help the dentist assess the tooth color. Selecting the correct color in dentistry can significantly impact the aesthetic outcome of dental treatments, and achieving the right shade is crucial for restorative and prosthodontic procedures like crowns, veneers, and dentures. However, this old method is dependent on the observer, the light, and the environment; therefore the dentists called for the use of advanced shade guides and digital technologies or instrumental methods. Recently, dentists needed to evaluate the whitening procedures over time to check the efficacy of the treatment tailored to that individual patient. Therefore, they asked for an accurate and reliable method to score and compare the differences in whitening by new tools. To perform this assessment, it is mandatory to evaluate the same area of the tooth at different times with a dedicated device and/or procedure [1, 2]. According to these needs, the tooth color can also be expressed by the WIO and the WI_D_ indexes, calculated from the instrumental colorimetry values internationally used for color evaluation [3]. In fact, these indexes were developed to determine overall whitening perception and to measure the difference in the teeth's whiteness after whitening treatments [4–6]. They were first scored using noncontact instrumental systems. Among them, one of the most cited was the digital imaging system (DIS), which gives images of teeth employing a Spectroradiometer Minolta CS-1007 or a noncontact Kodak SLR/N 14Mpixel digital camera fitted with a 90 mm macro-Nikon lens [7]. The procedure was complicated, and volunteers had to wear cheek retractors and place their heads on a chin holder in a light cabinet with a set of CIE illuminants D65 placed at specific angles. The pictures were then manually processed with the magnetic lasso tool, isolating upper central incisors and elaborating the Red, Green, and Blue (RGB) colors with Adobe Photoshop software [5]. The RGB parameters were converted to *L*, *a*, *b*, and WIO. The DIS system was validated for daily variability and operator agreement with excellent results [7]. The color determined with this method can be influenced by different factors, involving the type of light in the place of measurement and the experience of the operators, underlining the need for new instrumental approaches [8, 9]. Portable instruments that a dental practitioner can easily use in a chair-side setting without needing multiple cameras, light cabinets, and Adobe Photoshop processing have now been developed. One is the Vita Easyshade Advance 4.0 (VITA Zahnfabrik, Bad Säckingen, Germany), a digital spectrophotometer that works as a colorimeter and reports the *L*, *a*, and *b* color coordinates. This contact instrument ensures fast and hygienic readings thanks to single-use plastic protections. It is also straightforward to employ with easy-to-use software and screen. The Vita Easyshade Advance 4.0 has already been used to evaluate the effect of resin infiltration on white spot lesions on artificial teeth and compare the color of vital or nonvital natural teeth and the impact of different whitening dentifrices after tooth bleaching [10–12]. Vita Easyshade Advance 4.0 has also been employed to assess chewing gum's efficacy in preventing stains on natural teeth in a short-term, forced staining protocol [13]. When the Vita Easyshade Advance 4.0 is used to determine the whiteness of tooth color before and after a whitening treatment, acquiring data from the same area of the tooth is crucial to avoid drawbacks affecting the method's reproducibility. For this scope, a silicone mask was developed to ensure that the same or different operators could always place the instrument reading tip in the same tooth position at different times. Recently, a new device, Rayplicker, became available on the market. This is the first instrument with a dedicated standardized autoclavable guide to apply on the tooth surface to protect the measurement from environmental light. Moreover, it can analyze, with one single shot, the color of almost all the vestibular surfaces with easy-to-use dedicated software for PCs to calculate the color CIELAB coordinates. The Rayplicker has a plastic guide, which might result in the same function as the silicone mask, even without interference with the real color of the tooth. For a dental practitioner, the main clinical application of both devices is to help the dental operator check the real color of the tooth to inform the dental technician correctly; however, they can also be used for all restorative procedures to pick the best matching color material and also to test over time the whitening procedures. As described above, the Vita Easyshade Advance 4.0 scored single spots of the tooth surface while the Rayplicker claims to score all the surface. The dental operator has more trouble doing multiple measures at the same point at different times than measuring all the surfaces every time. Therefore, clinically, the Rayplicker might also be easier for the clinical practitioner to use. In this research, we compared the reproducibility of the color assessment with three procedures suggested to evaluate whitening: Vita Easyshade Advance 4.0, with and without the silicone mask developed to place the spectrophotometer reading tip always in the same tooth position, and the Rayplicker used following its guidelines. Therefore, the objective of this study was to evaluate the reproducibility of the color assessment according to the three procedures, at the same time or before/after 2'30”. We chose to test the most difficult situation in checking the whitening differences; thus, this timing was according to the clinically published studies focused on the instant optical procedures [5, 6]. Under the Null Hypothesis (Ho), it was postulated that there were no differences between the two methods with the same device and between the two devices in scoring the color of the teeth at the same time or before and after 2′30″.

## 2. Methods

### 2.1. Study Population

A random sample of 20 participants was selected from a pool of dental assistants from various private offices in Tuscany (Italy). The study followed ethical principles as in the Declaration of Helsinki and approximate Good Clinical Practice guidelines [14]. No substance was given in this study, and all participants signed informed written consent. The procedure on volunteers only screened their tooth color, did not affect their oral environment, and reproduced the normal techniques employed daily for a dental visit. This study was approved by the ethical committee “U.S. Investigational Review Board, Inc.” with IRB Number U.S. IRB2022PVM/02.

To enter this study, the subjects had to sign an informed consent form, be between 18 and 60 years old, and have all four upper incisors sound without restoration or prosthesis. They had to report no allergy to silicone and sign the approved written consent.

### 2.2. Study Design

The study was designed as a randomized, single-centered, cross-over study. To compare the effect of the mask on the variability of multiple measurements of color coordinates on natural teeth, the intravariability in the instrumental values given by the Vita Easyshade Advance 4.0 device (with and without a silicone mask) and the Rayplicker at the same time or before and after 2'30” was compared. This timing was set according to the clinically published studies focused on the evaluation of the instant whitening tools [5, 6, 15]. A dedicated dental mask was prepared for each volunteer. At the visit, he/she underwent these procedures: (1) provide teeth brushing 2 h before the appointment, (2) be randomly assigned at the sequence of the visits/tests, and (3) have the teeth color measured with the Vita Easyshade Advance 4.0 using or not a customized mask or with the Rayplicker. The measure of tooth color was arranged to assess reproducibility and/or stability over time as detailed in the following paragraphs. The testing phase lasted ~24 weeks.

### 2.3. Instruments and Mask

The Vita Easyshade Advance 4.0 (VITA Zahnfabrik, Bad Säckingen, Germany) and the Rayplicker (BOREA DENTAL, Limoges Cedex, France) were purchased from the manufacturers. Silicone dental impression material for dental prostheses (Ormaplus Putty-green, Major Prodotti Dentali S.p.A., Dental, Moncalieri, Torino, Italy) was used with a plastic tray to form a mask for the upper incisors, following the manufacturer's directions for use. The two silicone pastes were mixed and placed on the upper dental arch with the tray. After its assessment, it was removed, cleaned with water, dried, and then sent to the dental laboratory to be prepared. This silicone impression was used as a mask after the dental laboratory made a precise hole for applying the tip of this device to the tooth surface, using a bur guided by the Vita Easyshade Advance 4.0 3D-printed replica tip in hard plastic. Silicone is one of the most used materials to take impressions in dental prostheses and mucosa because it gives an accurate replica of the teeth and is stable without deformation over time. These properties allow the use of the mask more times. This material is available in different colors, but in our trial, this was not an issue because the changes from the real tooth color resulting from the color of the silicone mask were the same for all the measures that we processed in this study. Therefore, the systematic shifts of the tooth color due to the silicone color used for the customized mask were considered irrelevant because this study was planned to evaluate the reproducibility of the measurements rather than the absolute color as in prosthodontics procedures.

The Vita Easyshade Advance 4.0 and the Rayplicker instruments efficiently deliver the tooth color coordinates in the CIELAB color space. *L*, *a*, and *b* values were used to calculate the whiteness WIO and WI_D_ indexes as described in the literature [3]. The Rayplicker measures a large area with a value for each single point; therefore, we had to calculate the mean of them with its dedicated software. Instead, the Vita Easyshade Advance 4.0 instrument measures a punctiform area and shows us the resulting mean values. We also evaluated the customized silicone mask's effect on the variability of tooth color measures with only the Vita Easyshade Advance 4.0. All measurements were performed on one incisor tooth of the human volunteers. The teeth' color measurements with and without the mask for all tests were scored using the Vita Easyshade Advance 4.0 and the Rayplicker instrument following the manufacturer's directions of use. The Rayplicker was always used without a customized mask but with its dedicated guide, which is similar in scope, however developed to score the real color of the tooth surface. The data were immediately available with the Vita Easyshade Advance 4.0; instead, due to the elaboration of the image, the process for the Rayplicker might require more than 20 s. Therefore, due to the different times of acquisition of data between the two devices, to perform acquisition experiments over comparable periods, we had to score fewer measures with the Rayplicker than with the Vita Easyshade Advance 4.0.

### 2.4. Color Measurements

To reduce the potential bias related to the influence introduced by different operators, all the measurements were conducted by a single, expert, trained operator who followed a standardized measurement protocol. In the first test, the same operator did 15 consecutive readings with the Vita Easyshade Advance 4.0 in the shortest possible time on 20 volunteers, without wearing the mask; then, after a pause of at least 10 min, the same operator applied their customized masks to the same healthy volunteers and took 15 consecutive readings; after at least 10 min, the same operator made three straight readings with the Rayplicker using the suppliers' guide and in the shortest possible time on the same 20 volunteers. Both instruments were used after their calibration procedures as suggested by their manufacturers. The objective of this test was to assess the dispersion of the measurements. In a second test, the same operator acquired triplicate measures for the Vita Easyshade Advance 4.0 with the mask and duplicate measures for the Rayplicker with the supplier guide on the natural teeth of 20 volunteers at time 0 and after 2'30”. The mask was removed during the waiting time for the second measurement. At least 10 min of washout time were set between the tests with the two devices. For the first test, the primary outcome was the dispersion of measures scored with three methods: Vita Easyshade Advance 4.0 with the silicon customized mask, Vita Easyshade Advance 4.0 without the mask, and Rayplicker with the supplier guide. The endpoints were the intrasubject variances and the intrasubject percent coefficients of variation (%CV) of measures of the score of the tooth color (CIELAB) and the whitening indexes (WIO and Wl_D_) among the devices and the procedures. For the second test, the primary outcome was the difference between the measurements taken before and after 2'30” with two methods: Vita Easyshade Advance 4.0 with the customized mask and Rayplicker with its guide.

### 2.5. Statistical Analysis

The primary study objectives were to compare variabilities with in-subject results among devices and procedures. Two outcome measures were considered: variance and coefficient of variation (CV). Differences in variances were tested using the Bartlett test, while a comparison of %CV was performed with parametric and sign nonparametric tests for paired data, when appropriate. The observations from our previous similar preliminary study suggested to include about 20 subjects; however, no similar studies have been published; thus, we chose to calculate power and sample size considering standardized differences (means difference divided by their standard deviation) [16, 17]. With 20 participants per group, as suggested above, we can exclude an effect size (standardized difference, Cohen *D*) >0.5 which is considered a medium effect size at the significance level of 0.05 with one side, with the 80% power when the effect size is 0 (no differences among devices). Therefore, the power calculations indicated that setting this number to 20 subjects would yield an 80% power to detect a significant difference at an alpha level of 0.05. The data were analyzed using version 9.4 of the SAS System for Windows 2016 (SAS Institute lnc., Cary, NC, USA).

## 3. Results

Twenty healthy adult volunteers who met the inclusion criteria entered the study. There were 7 men and 13 women aged between 22 and 59 (mean 35.9). In the first test, the color of each tooth was measured 15 consecutive times with and without the mask using the Vita Easyshade Advance 4.0 instrument and three successive times with the Rayplicker. As expected, the color of the mask significantly changed the score of the real tooth color, but it did not influence the evaluation of the differences among the measurements, as explained previously. However, in this specific situation, our customized silicone mask was green, causing in raising the *L* values, lowering the *a* values, and increasing the *b* values.

The results indicated that, for each device, the intrasubject means, the variance, and the %CV, were minimal, demonstrating high reproducibility. Bartlett test for homogeneity of variance resulted in statistically significant differences only for the *a* score (*p*  < 0.001), with a greater variance for the Vita Easyshade Advance 4.0 with the mask compared with either the Vita Easyshade Advance 4.0 without the mask and the Rayplicker. The evaluation of %CV is shown in Table 1. The %CV standardizes variability according to the mean values, offering a clearer interpretation of the comparison results. The Vita Easyshade Advance 4.0 without a mask had a significantly greater CV for *L* and *b* scores, while the Vita Easyshade Advance 4.0 with the mask and the Rayplicker appeared similar for all the scores.

The analysis of the location and dispersion statistics of the second test for the evaluation with Vita Easyshade Advance 4.0 with the customized silicon mask at time 0 and after 2′32″ showed complete reproducibility in variance and %CV data. The evaluation of %CV is shown in Table 2. No difference in %CV at different time values has been detected. The analysis of the location and dispersion statistics for evaluation with Rayplicker at time 0 and after 2′32″ showed complete reproducibility in variance and %CV data. The evaluation of %CV is shown in Table 3. *p* Values associated with the Bartlett test for homogeneity of variance executed for each one of the above instruments resulted in >0.90 for all the score comparisons at time 0 and after 2′32″. No difference in %CV values at different times has been detected. The variance and the %CV resulted in minimal within each score. Bartlett's test for homogeneity of variance indicated that the measures done with the Vita Easyshade Advance 4.0 without using the customized mask had a significantly greater CV for *L* and *b* scores than those obtained using the Vita Easyshade Advance 4.0 with the mask or the Rayplicker. The comparison of the values obtained before and after 2′30” indicated complete reproducibility in variance and %CV data.

## 4. Discussion

This study suggested that the use of a custom-made silicone mask with the Vita Easyshade Advance 4.0 device can reduce the variability of the optical parameters of the color scored on tooth surfaces in vivo and that the Rayplicker has similar performances to the Vita Easyshade Advance 4.0 with a customized silicone mask. Because environmental situations and operator procedures largely affect optical parameters, it was planned to reduce this bias by creating a guide for the tip of the Vita Easyshade Advance 4.0 instrument to drive it on the same area of the surface of the tooth and protect the measure from the different illumination conditions. This method allows the operator to measure the color of the same spot on the tooth surface at different times, evaluating the differences in whitening. In dental practice, it was empirically observed that the angle between the reading tip of the Vita Easyshade Advance 4.0 instrument, and the tooth surface can influence the *L*, *a*, and *b* values. Since it is hard to reproduce the same angle at different visits or pre- and post-treatment with a whitening agent like toothpaste or chewing gum, and tooth color may not be uniform across the whole surface, it was hypothesized that a custom-made mask with a hole fitting the tip and neck of the Vita Easyshade Advance 4.0 instrument could ensure more stable readings. The color of the mask influenced the *L*, *a*, and *b* absolute values due to reflection from the material, but this was not the focus of our study, which was planned to assess the reproducibility of the measurements and not to score the real color as it is the case in dental practice, especially for prosthodontic procedures.

The Rayplicker was introduced on the market to avoid this problem due to the large portion of the tooth surface that it can scan with its dedicated autoclavable guide to be applied on the tooth. This device has a dedicated calibration tip, which is positioned (clipping) at the end of the measuring head. Thanks to its specific design, it allows to take support on the patient's teeth, thus ensuring a precise positioning of the device to score the same area at different times. This procedure requires more time when compared to the Vita Easyshade Advance 4.0 because the Rayplicker must be connected to the PC with its dedicated software to analyze the image, but this method can score the true color of the dental surface scanned and, therefore, it could be used by the dentist not only for the evaluation of the whitening tool but also for prosthodontic procedures. In this study, we checked the variability of the measures taken with and without the customized silicon mask for the Vita Easyshade Advance 4.0 and the Rayplicker with its guide and the repeatability at different times. These tests aimed to validate a method to compare two measures taken on the same tooth surface at different times for the two most important and published whitening indexes WIO and WI_D_. Therefore, in this study, the time between the readings was compatible with the time needed to assess volunteers before and after treatment with an optical whitening toothpaste used for 1'30” or one chewing gum for 2′32″ [5, 6, 15]. Measures obtained at 2′32″ minutes did not show a clinical difference for the means for the color coordinates *L*, *a*, *b*, WIO, and WI_D,_ which are the most important parameters for testing the whitening.

Therefore, dental hygienists could objectively assess the effectiveness of the whitening treatment via the change in the WIO and WI_D_ indices, being reassured that there is no time drift in the indices due to the reading method. Recently, there has been growing interest in whitening, with many studies designed with different indexes and perceptibility thresholds [18, 19]. The optical whitening properties of toothpaste were recently measured using the same portable instrument employed in this study but with a custom-made spacer to direct the reading tip to a reproducible area of the tooth [20]. Unfortunately, no further details are available about this spacer, so we could not compare it with the mask we used or the Rayplicker. However, in that study, all measures were done in triplicate as in this research, but we employed for the first time a modified method based on a contact instrument (Vita Easyshade Advance 4.0) and a guide mask, which ensured excellent repeatability; still, the longer time of acquisition for the noncontact instrument (Rayplicker) hampers repeated measurements in a short period. In our pilot study, we only evaluated the use of the Vita Easyshade Advance 4.0 with and without the mask, and our preliminary results agreed with the findings of this trial, suggesting that the use of an individual silicone mask improved the precision of a portable digital spectrophotometer device for multiple measurements of *L*, *a*, *b* and WIO, WI_D_ [16]. Researchers do not agree on the best method to determine the tooth color in prosthodontics and/or in whitening evaluation. Brand et al. suggested that an intraoral scanner (TriosColor) can be a good alternative to the current standard of visual tooth color determination and comparable to the Vita Easyshade Advance 4.0 that they assumed as the reference instrument. Instead, Ratzmann et al. stated that the 3D system can be favorable in bleaching studies owing to the added bleaching shades but may confuse human raters and even electronic devices [21, 22]. These methods are more expensive and require adequate training for the operator and a long time for image acquisition; thus, they are suggested for complex prosthodontic rehabilitation and not for easy whitening checking by the dental hygienists. The main limitations of this study are the limited number of participants and measurements, and the relatively homogenous sample consisting of healthy dental assistants without restorations or discolorations that may not reflect the broader patient population, even if it can improve compliance. We did not test different complex dental conditions and different kinds of patients, and the sample size could not be assessed from previous data published except for our similar pilot study; therefore, to generalize our results and observations, they must be confirmed by further investigations. This method of checking and comparing the tooth color over time using a dedicated silicone mask cannot be suggested for clinical routine applications by practitioners in their patients. It can be useful for researchers to evaluate the whitening effect of a treatment in volunteers. Instead, the use of Rayplicker, which showed similar reliability, can be suggested for all clinical applications, such as whitening procedures and prosthetic color matching because it is easy to use and the color scored is the real color of the tooth. Summarizing, the use of a silicone mask with the Vita Easyshade Advance 4.0 must be intended only for the scientific evaluation of the whitening over time with a tool and not to measure the real color of the tooth; otherwise, the use of the Rayplicker could be extended to all clinical procedures.

## 5. Conclusions

This study indicates that the silicone mask improves the precision of the Vita Easyshade Advance 4.0, and the Rayplicker can be a valid alternative for studies measuring the changes in tooth color after whitening treatments.

## 6. Clinical Relevance

### 6.1. Scientific Rationale for the Study

Dental practitioners and hygienists generally use a portable digital spectrophotometer to assess and compare over time the tooth color. This procedure requires that the device should score the same precise area of the tooth surface, and this is the main critical factor in reproducibility.

### 6.2. Principal Findings

The use of customized silicone masks improves the precision of Vita Easyshade and the new Rayplicker results similarly.

### 6.3. Practical Implications

The use of the Rayplicker or the Vita Easyshade with a mask can be very useful for researchers, dentists, and hygienists to measure the changes in tooth whitening after any treatment.

## Figures and Tables

**Table 1:** Evaluation of % coefficient of variation (CV) difference intrasubjects (adjustment with Tukey–Kramer).

	Tool 1	Tool 2	%CV difference	Standard error	*p*-Value	Adjusted *p*-Value
L_CV	Easyshade no mask	Easyshade with mask	0.56	0.11	<0.0001	<0.0001
L_CV	Easyshade no mask	Rayplicker	0.45	0.11	0.0002	0.0005
L_CV	Easyshade with mask	Rayplicker	−0.11	0.11	0.3209	0.5778
a_CV	Easyshade no mask	Easyshade with mask	80.62	51.55	0.1261	0.2734
a_CV	Easyshade no mask	Rayplicker	14.28	51.55	0.7832	0.9586
a_CV	Easyshade with mask	Rayplicker	−66.34	51.55	0.2059	0.4111
b_CV	easyshade no mask	Easyshade with mask	1.93	0.28	<0.0001	<0.0001
b_CV	easyshade no mask	Rayplicker	1.23	0.28	<0.0001	0.0003
b_CV	Easyshade with mask	Rayplicker	−0.69	0.28	0.0187	0.048
WI_D__CV	Easyshade no mask	Easyshade with mask	14.86	11.23	0.1935	0.391
WI_D__CV	Easyshade no mask	Rayplicker	13.58	11.23	0.2338	0.4548
WI_D__CV	Easyshade with mask	Rayplicker	−1.28	11.23	0.9098	0.9929
WIO_CV	Easyshade no mask	Easyshade with mask	6.76	3.65	0.072	0.1672
WIO_CV	Easyshade no mask	Rayplicker	6.43	3.65	0.0861	0.1959
WIO_CV	Easyshade with mask	Rayplicker	−0.32	3.65	0.9301	0.9957

**Table 2:** % Coefficient of variation (CV) difference evaluation over time with the Vita Easyshade Advance 4.0 with the customized silicone mask (effect pre–post: pre at time 0 and post at time 2′30″).

	%CV difference	Standard error	*T*-value	*p*-Value
L_CV	−0.02	0	−0.78	0.44
a_CV	−0.46	1	−0.81	0.43
b_CV	0.07	0	1.03	0.32
WI_D__CV	0.30	1	0.47	0.64
WIO_CV	−1.02	1	−1.19	0.25

**Table 3:** % Coefficient of variation (CV) evaluation over time with the Rayplicker (effect pre–post: pre at time 0 and post at time 2′30″).

	%CV estimate	Standard error	*T*-value	*p*-Value
L_CV	0.06	0.05	1.26	0.22
a_CV	0.69	12.33	0.06	0.96
b_CV	0.01	0.29	0.03	0.98
WI_D__CV	0.49	0.63	0.78	0.44
WIO_CV	−1.46	1.31	−1.11	0.28

## Data Availability

The whole data that support the findings of this study are available from the corresponding author upon request.
